# Genome-wide association study of sensory disturbances in the inferior alveolar nerve after bilateral sagittal split ramus osteotomy

**DOI:** 10.1186/1744-8069-9-34

**Published:** 2013-07-08

**Authors:** Daisuke Kobayashi, Daisuke Nishizawa, Yoshito Takasaki, Shinya Kasai, Takashi Kakizawa, Kazutaka Ikeda, Ken-ichi Fukuda

**Affiliations:** 1 Department of Oral Health and Clinical Science, Division of Dental Anesthesiology, Orofacial Pain Center, Suidoubashi Hospital, Tokyo Dental College, 2-9-18 Misaki-cho, Chiyoda-ku, Tokyo 101-0061, Japan; 2 Addictive Substance Project, Tokyo Metropolitan Institute of Medical Science, 2-1-6 Kamikitazawa, Setagaya-ku, Tokyo 156-8506, Japan; 3 Department of Dentistry and Oral surgery, Tokyo Metropolitan Tama Medical Center, 2-8-29 Musashidai, Fuchu-shi, Tokyo 183-8524, Japan; 4 Department of Dentistry and Oral Surgery, National Hospital Organization, Takasaki General Medical Center, 36 Takamatsu-Cho, Takasaki-shi, Gunma 370-0829, Japan; 5 Department of Oral Health and Clinical Science, Division of Oral and Maxillo-facial Surgery, Tokyo Dental College, 2-9-18 Misaki-cho, Chiyoda-ku, Tokyo 101-0061, Japan

**Keywords:** Bilateral sagittal split ramus osteotomy, Hypoesthesia, Dysesthesia, Neuropathic pain, Genome-wide association study

## Abstract

**Background:**

Bilateral sagittal split ramus osteotomy (BSSRO) is a common orthognatic surgical procedure. Sensory disturbances in the inferior alveolar nerve, including hypoesthesia and dysesthesia, are frequently observed after BSSRO, even without distinct nerve injury. The mechanisms that underlie individual differences in the vulnerability to sensory disturbances have not yet been elucidated.

**Methods:**

The present study investigated the relationships between genetic polymorphisms and the vulnerability to sensory disturbances after BSSRO in a genome-wide association study (GWAS). A total of 304 and 303 patients who underwent BSSRO were included in the analyses of hypoesthesia and dysesthesia, respectively. Hypoesthesia was evaluated using the tactile test 1 week after surgery. Dysesthesia was evaluated by interview 4 weeks after surgery. Whole-genome genotyping was conducted using Illumina BeadChips including approximately 300,000 polymorphism markers.

**Results:**

Hypoesthesia and dysesthesia occurred in 51 (16.8%) and 149 (49.2%) subjects, respectively. Significant associations were not observed between the clinical data (i.e., age, sex, body weight, body height, loss of blood volume, migration length of bone fragments, nerve exposure, duration of anesthesia, and duration of surgery) and the frequencies of hypoesthesia and dysesthesia. Significant associations were found between hypoesthesia and the rs502281 polymorphism (recessive model: combined *χ*^*2*^ = 24.72, nominal *P* = 6.633 × 10^-7^), between hypoesthesia and the rs2063640 polymorphism (recessive model: combined *χ*^*2*^ = 23.07, nominal *P* = 1.563 × 10^-6^), and between dysesthesia and the nonsynonymous rs2677879 polymorphism (trend model: combined *χ*^*2*^ = 16.56, nominal *P* = 4.722 × 10^-5^; dominant model: combined *χ*^*2*^ = 16.31, nominal *P* = 5.369 × 10^-5^). The rs502281 and rs2063640 polymorphisms were located in the flanking region of the *ARID1B* and *ZPLD1* genes on chromosomes 6 and 3, whose official names are “AT rich interactive domain 1B (SWI1-like)” and “zona pellucida-like domain containing 1”, respectively. The rs2677879 polymorphism is located in the *METTL4* gene on chromosome 18, whose official name is “methyltransferase like 4”.

**Conclusions:**

The GWAS of sensory disturbances after BSSRO revealed associations between genetic polymorphisms located in the flanking region of the *ARID1B* and *ZPLD1* genes and hypoesthesia and between a nonsynonymous genetic polymorphism in the *METTL4* gene and dysesthesia.

## Background

Neuropathic pain in the orofacial region is a clinical manifestation of trigeminal nerve injury following oral surgery. Neuropathic pain subsequent to nerve damage at a central or peripheral site remains a major problem for both patients and clinicians because the pain is usually extremely intense and often refractory to various conventional pain therapies. Moreover, remarkable individual differences in the vulnerability to neuropathic pain exist. Many studies have been performed to reveal the mechanisms that underlie neuropathic pain, but only a few genetic studies have focused on neuropathic pain [1,2], possibly because individual differences across patients with neuropathic pain are usually affected by various factors other than genetic factors.

Sensory disturbances, including hypoesthesia and dysesthesia, often appear as a prodromal symptom of neuropathic pain. Sensory disturbances or neuropathic pain in the inferior alveolar nerve are inevitably caused by a primary lesion or dysfunction of the nerve. The symptoms, however, are subject to individual differences in daily clinical practice and may be related to genetic factors. Bilateral sagittal split ramus osteotomy (BSSRO) is commonly conducted to correct jaw deformities, such as mandibular prognathism. Sensory disturbances in the inferior alveolar nerve, including hypoesthesia and dysesthesia, are frequently observed in the lower lip and mental area after BSSRO, even without distinct nerve injury. Symptom frequency 1 or 2 weeks after BSSRO is reported in 25-56% of patients [3-5]. Considering that almost all patients who undergo BSSRO are young and healthy and the degree of surgical invasiveness, surgical site, and surgical procedures are highly consistent across cases, environmental factors appear to have relatively little impact on individual differences in the vulnerability to sensory disturbances or neuropathic pain after BSSRO.

Innovative techniques have been used to investigate the genetic factors related to various human traits. A wide array of information on the entire human genome has accumulated, and the results of genome-wide association studies (GWASs) have been reported [6,7]. A marked increase in the rate of discovery of genes associated with various diseases has also occurred [8].

The present GWAS investigated the relationships between genetic polymorphisms and the vulnerability to sensory disturbances after BSSRO.

## Results

### Clinical data overview and SNP data management for GWAS

Hypoesthesia and dysesthesia occurred in 51 (16.8%) and 149 (49.2%) of the 304 and 303 patients, respectively (Table 1). Logistic regression analysis revealed no significant associations between the clinical data and frequency of hypoesthesia or dysesthesia after BSSRO (data not shown).

**Table 1:** Expression frequency of hypoesthesia and dysesthesia after BSSRO

	**Normal**	**Abnormal**
Hypoesthesia	253 (83.2%)	51 (16.8%)
Dysesthesia	154 (50.8%)	149 (49.2%)

After filtering the markers by genotype call frequency, “Cluster sep”, and minor allele frequencies in the first quality control assessment of the genotyping data, 243,501 markers were selected. These merged genotype data from five different BeadChips consisted of single nucleotide polymorphism (SNP) markers on the autosome or sex chromosome, and no mitochondrial marker was included. Furthermore, 272 markers were excluded based on the Hardy-Weinberg equilibrium test (*P* ≤ 2 × 10^-7^). As a result, a total of 243,229 SNP markers (including 4,822 nonsynonymous SNPs) were selected for the subsequent association study (Additional file 1: Figure S1 and Additional file 2: Figure S2).

### GWAS identified several loci associated with sensory disturbances in the inferior alveolar nerve after BSSRO

The GWAS was performed to detect any signals associated with hypoesthesia or dysesthesia after BSSRO as three-stage analyses for two independent patterns: (1) a normal GWAS procedure that targeted all of the SNPs that were available (Additional file 1: Figure S1) and (2) a GWAS procedure that targeted only nonsynonymous SNPs that tended to affect the function of the protein encoded by the relevant gene (Additional file 2: Figure S2).

In the first analysis that targeted all of the SNPs, six, five, and 22 SNPs were selected as the top candidates associated with hypoesthesia for the trend, dominant, and recessive models for each minor allele, respectively, after the final stage (Table 2). Seven, four, and nine SNPs were selected as the top candidates associated with dysesthesia for the trend, dominant, and recessive models for each minor allele, respectively, after the final stage (Table 3). Among these, two SNPs, rs502281 and rs2063640, showed significant associations with hypoesthesia after the final stage in the recessive model (rs502281: *χ*^*2*^ = 16.44, *Q* = 0.0196; rs2063640: *χ*^*2*^ = 14.38, *Q* = 0.0291; Table 2). None of the SNPs showed significant associations with dysesthesia after the final stage in any of the models (Table 3).

**Table 2:** Top candidate SNPs selected after final stage analysis in 3-stage GWAS targeting all SNPs (hypoesthesia)

**Model**	**Rank**	**SNP**	**CHR**	**Position**	**1st stage**		**2nd stage**		**Final stage**			**Combined**		**Genotype**		**Related gene**
					***X***^***2***^	***P***	***X***^***2***^	***P***	***X***^***2***^	***P***	***Q***	***X***^***2***^	***P***	**Abnormal**	**Normal**	
Trend	1	rs7228266	18	40874531	4.377	0.0364	6.005	0.0143	9.102	0.0026	0.567	19.15	1.21E-05	7/28/16	8/92/153	*SETBP1*
Trend	2	rs6537883	1	110206794	5.962	0.0146	5.917	0.015	6.385	0.0115	0.6855	18.13	2.06E-05	2/6/43	35/93/121	*CSF1*
Trend	3	rs9474312	6	52706460	5.95	0.0147	6.026	0.0141	5.866	0.0154	0.6855	18.03	2.18E-05	9/24/18	11/84/157	*LOC730152*
Trend	4	rs1870761	11	122356773	4.184	0.0408	7.947	0.0048	4.011	0.0452	0.7696	15.87	6.79E-05	0/8/42	16/100/134	*BSX*
Trend	5	rs139131	22	42912379	7.103	0.0077	4.071	0.0436	4.38	0.0364	0.7696	15.6	7.81E-05	0/7/44	10/100/143	*PARVG*
Trend	6	rs2295343	20	3683601	4.657	0.0309	4.81	0.0283	6.327	0.0119	0.6855	15.11	0.000101	0/6/45	10/93/150	*C20orf27*
Dominant	1	rs6537883	1	110206794	7.505	0.0062	5.984	0.0144	8.612	0.0033	0.4897	21.79	3.04E-06	2/6/43	35/93/121	*CSF1*
Dominant	2	rs139131	22	42912379	7.32	0.0068	4.126	0.0422	4.264	0.0389	0.763	15.87	6.78E-05	0/7/44	10/100/143	*PARVG*
Dominant	3	rs2295343	20	3683601	4.574	0.0325	5.147	0.0233	6.631	0.01	0.5288	15.46	8.41E-05	0/6/45	10/93/150	*C20orf27*
Dominant	4	rs10502849	18	40866089	5.286	0.0215	3.933	0.0473	6.161	0.0131	0.5288	15.25	9.41E-05	11/28/11	20/101/1	*SETBP1*
Dominant	5	rs707816	6	13742961	4.586	0.0322	3.933	0.0473	5.075	0.0243	0.7257	13.08	0.000299	2/15/34	33/121/99	*RANBP9*
Recessive	1	rs2817461	6	156954704	8.06	0.0045	12.55	0.0004	12.53	0.0004	0.0521	30.33	3.64E-08	9/42	3/250	*ARID1B*
Recessive	2	rs502281	6	156910640	3.991	0.0458	6.935	0.0085	16.44	5E-05	0.0196*	24.72	6.63E-07	7/9/35	2/71/180	*ARID1B*
Recessive	3	rs2063640	3	103685735	6.085	0.0136	4.932	0.0264	14.38	0.0001	0.0291*	23.07	1.56E-06	15/11/25	17/110/125	*ZPLD1*
Recessive	4	rs13236243	7	17284837	10.43	0.0012	6.658	0.0099	4.14	0.0419	0.7421	21.14	4.28E-06	16/16/19	21/121/111	*LOC729939*
Recessive	5	rs1054611	12	10061428	6.1	0.0135	4.344	0.0371	11.07	0.0009	0.0775	20.61	5.64E-06	10/16/25	8/84/161	*CLEC12B*
Recessive	6	rs6833812	4	5161041	3.991	0.0458	12.25	0.0005	5.428	0.0198	0.4066	20.11	7.32E-06	4/9/38	0/57/196	*STK32B*
Recessive	7	rs1059513	12	55775976	8.06	0.0045	6.062	0.0138	5.428	0.0198	0.4066	20.11	7.32E-06	4/8/39	0/32/221	*STAT6*
Recessive	8	rs1998930	6	156945948	5.157	0.0232	11.29	0.0008	6.002	0.0143	0.4066	19.52	9.94E-06	22/21/8	40/130/83	*ARID1B*
Recessive	9	rs4235662	5	84203580	7.754	0.0054	6.062	0.0138	5.428	0.0198	0.4066	19.42	1.05E-05	5/18/28	1/96/156	*EDIL3*
Recessive	10	rs3804357	4	102221146	5.165	0.0231	6.551	0.0105	10.84	0.001	0.0775	19.3	1.12E-05	8/16/27	5/102/144	*PPP3CA*
Recessive	11	rs4732828	8	28050160	3.991	0.0458	5.99	0.0144	5.791	0.0161	0.4066	15.27	9.31E-05	3/5/42	0/20/232	*ELP3*
Recessive	12	rs4658506	1	240012540	3.991	0.0458	6.062	0.0138	5.428	0.0198	0.4066	15.03	0.000106	3/11/37	0/67/186	*WDR64*
Recessive	13	rs2868145	19	37738954	3.991	0.0458	6.062	0.0138	5.428	0.0198	0.4066	15.03	0.000106	3/10/38	0/42/211	*PDCD5*
Recessive	14	rs1564492	15	71720771	3.991	0.0458	6.062	0.0138	5.428	0.0198	0.4066	15.03	0.000106	3/10/38	0/37/216	*NPTN*
Recessive	15	rs1072056	5	110532014	3.991	0.0458	6.062	0.0138	5.428	0.0198	0.4066	15.03	0.000106	3/6/42	0/59/194	*WDR36*
Recessive	16	rs10512369	9	109805180	3.991	0.0458	6.062	0.0138	5.428	0.0198	0.4066	15.03	0.000106	3/7/41	0/25/228	*LOC392382*
Recessive	17	rs10841907	12	21942563	5.185	0.0228	5.026	0.025	4.956	0.026	0.5072	14.95	0.00011	11/18/21	14/104/135	*ABCC9*
Recessive	18	rs9942977	9	108422182	3.895	0.0484	6.062	0.0138	5.428	0.0198	0.4066	14.91	0.000113	3/5/43	0/28/223	*LOC644620*
Recessive	19	rs395640	21	26891730	3.991	0.0458	6.062	0.0138	6.074	0.0137	0.4066	14.55	0.000136	4/12/35	1/75/177	*CYYR1*
Recessive	20	rs13110230	4	178153868	3.991	0.0458	6.062	0.0138	6.074	0.0137	0.4066	14.55	0.000136	4/9/38	1/48/204	*VEGFC*
Recessive	21	rs1960997	11	97149034	3.991	0.0458	4.344	0.0371	5.675	0.0172	0.4066	11.66	0.000638	6/19/26	5/104/144	*CNTN5*
Recessive	22	rs9535720	13	51092945	3.999	0.0455	3.907	0.0481	4.141	0.0419	0.7421	11.61	0.000656	6/19/26	38/140/75	*WDFY2*

CHR, chromosome number; Position, chromosomal position (bp); *Q*, *Q* value for FDR correction of multiple comparison; Related gene, the nearest gene from the SNP site; *, Significant after FDR correction (*Q* < 0.05).

**Table 3:** Top candidate SNPs selected after final stage analysis in 3-stage GWAS targeting all SNPs (dysesthesia)

**Model**	**Rank**	**SNP**	**CHR**	**Position**	**1st stage**		**2nd stage**		**Final stage**			**Combined**		**Genotype**		**Related gene**
					***X***^***2***^	***P***	***X***^***2***^	***P***	***X***^***2***^	***P***	***Q***	***X***^***2***^	***P***	**Abnormal**	**Normal**	
Trend	1	rs6829274	4	36167210	4.852	0.0276	6.571	0.0104	5.444	0.0196	0.6536	16.91	3.91E-05	12/65/72	29/83/42	*FLJ16686*
Trend	2	rs945877	1	197785628	6.571	0.0104	4.92	0.0266	4.828	0.028	0.6536	16.84	4.07E-05	45/74/30	24/69/61	*LOC647202*
Trend	3	rs2677879	18	2537500	4.078	0.0435	6.071	0.0137	6.585	0.010	0.6536	16.56	4.72E-05	13/51/84	28/73/51	*METTL4*
Trend	4	rs7825569	8	70057575	5.909	0.0151	6.756	0.0093	3.846	0.0499	0.7411	15.31	9.14E-05	42/77/30	21/76/57	*C8orf34*
Trend	5	rs1064108	14	64470018	4.777	0.0288	5.124	0.0236	4.78	0.0288	0.653	15.01	0.000107	31/63/55	8/66/80	*CHURC1*
Trend	6	rs11817730	10	9934850	4.651	0.031	3.85	0.0498	6.73	0.0095	0.6536	14.48	14.48	1/13/135	4/37/113	*C10orf65*
Trend	7	rs12603925	17	14929712	4.248	0.0393	4.005	0.0454	4.268	0.0388	0.7411	12.29	0.000456	20/66/62	39/74/38	*LOC44178*
Dominant	1	rs2210585	20	10077600	7.653	0.0057	4.356	0.0369	6.442	0.0111	0.816	17.94	2.28E-05	24/90/35	15/67/72	*SNAP25*
Dominant	2	rs2677879	18	2537500	3.905	0.0481	5.79	0.0161	6.669	0.0098	0.816	16.31	5.37E-05	13/51/84	28/73/51	*METTL4*
Dominant	3	rs10805209	4	8600745	6.282	0.0122	4.376	0.0365	4.474	0.0344	0.816	13.72	0.000212	28/68/53	47/81/26	*GPR78*
Dominant	4	rs6477523	9	108304897	4.762	0.0291	4.356	0.0369	4.151	0.0416	0.816	13.59	0.000228	25/89/35	32/55/67	*LOC644620*
Recessive	1	rs1567375	11	119007687	5.911	0.0151	9.524	0.002	4.67	0.0307	0.4279	19	1.31E-05	35/67/47	9/81/64	*PVRL1*^✝^
Recessive	2	rs4902304	14	64189429	9.896	0.0017	4.376	0.0365	4.149	0.0417	0.4279	17.32	3.15E-05	10/79/60	37/67/50	*PLEKHG3*
Recessive	3	rs6982411	8	135076849	6.562	0.0104	5.275	0.0216	3.977	0.0461	0.4279	15.69	7.46E-05	17/52/80	1/58/95	*LOC729395*
Recessive	4	rs730545	5	180446073	4.072	0.0436	4.057	0.044	7.119	0.0076	0.3997	15.55	8.05E-05	18/96/34	47/64/41	*BTNL9*
Recessive	5	rs10837504	11	40775682	4.595	0.0321	3.852	0.0497	5.934	0.0149	0.4279	14.19	0.000165	2/70/77	19/59/76	*LRRC4C*
Recessive	6	rs7551844	1	53833921	5.176	0.0229	4.631	0.0314	3.916	0.0478	0.4279	13.7	0.000214	20/89/40	48/70/36	*GLIS1*
Recessive	7	rs236008	16	6981244	4.062	0.0439	4.174	0.0411	5.273	0.0217	0.4279	13.43	0.000248	15/49/85	1/63/90	*HYDIN*
Recessive	8	rs2838271	21	43586302	4.595	0.0321	4.174	0.0411	4.362	0.0368	0.427	13.15	0.000288	2/58/89	18/56/80	*LOC727743*
Recessive	9	rs10497603	2	183044713	4.594	0.0321	4.019	0.045	3.915	0.0479	0.4279	12.08	0.000511	16/55/78	2/67/85	*PDE1A*

CHR, chromosome number; Position, chromosomal position (bp); *Q*, *Q* value for FDR correction of multiple comparison; Related gene, the nearest gene from the SNP site; ^✝^, modified from the Illumina annotation file.

In the second analysis that targeted nonsynonymous SNPs, four, three, and 14 SNPs were selected as the top candidates associated with hypoesthesia for the trend, dominant, and recessive models for each minor allele, respectively, after the second stage (Table 4). Three, five, and two SNPs were selected as the top candidates associated with dysesthesia, respectively, after the second stage (Table 5). Among these, none of the SNPs showed significant associations with hypoesthesia after the final stage in any of the models (Table 4). One SNP, rs2677879, showed significant associations with dysesthesia after the final stage in the trend and dominant models (trend model: *χ*^*2*^ = 6.585, *Q* = 0.0309; dominant model: *χ*^2^ = 6.669, *Q* = 0.0491; Table 5). Statistical power analyses revealed that the expected power (1 minus type II error probability) was only 19.5% and 15.1% for the Cohen’s conventional “small” effect size of 0.10 [9] and 90.8% and 84.6% for the medium effect size of 0.30, with a total of 120 valid samples in each stage. The degrees of freedom were set at 1 and 2, respectively, for the nominal type I error probability of 0.05. The estimated effect sizes were 0.26 and 0.28 to achieve 80% power for this type I error probability using our samples. The degrees of freedom were set at 1 and 2, respectively.

**Table 4:** Top candidate SNPs selected after second stage analysis in 3-stage GWAS targeting nonsynonymous SNPs (hypoesthesia)

**Model**	**Rank**	**SNP**	**CHR**	**Position**	**1st stage**		**2nd stage**		**Final stage**			**Combined**		**Genotype**		**Related gene**
					***X***^***2***^	***P***	***X***^***2***^	***P***	***X***^***2***^	***P***	***Q***	***X***^***2***^	***P***	**Abnormal**	**Normal**	
Trend	1	rs2839227	21	46610952	5.771	0.0163	8.95	0.0028	0.4962	0.4812	0.7406	11.63	0.00065	7/29/15	19/88/143	*PCNT*
Trend	2	rs4074536	1	116112490	4.724	0.0298	3.904	0.0482	0.1467	0.7017	0.7406	7.338	0.006753	6/24/21	58/135/60	*CASQ2*
Trend	3	rs2296351	13	51607939	4.131	0.0421	4.244	0.0394	0.1096	0.7406	0.7406	6.061	0.01382	2/19/30	5/56/192	*NEK3*
Trend	4	rs1339847	1	246105917	4.141	0.0419	4.938	0.0263	0.7541	0.3852	0.7406	3.974	0.04622	6/11/34	4/65/184	*TRIM58*
Dominant	1	rs2228576	12	6327323	7.136	0.0076	4.904	0.0268	3.013	0.0826	0.2477	15.42	8.6E-05	9/35/6	37/108/102	*SCNN1A*
Dominant	2	rs2839227	21	46610952	7.313	0.0068	5.538	0.0186	1.604	0.2053	0.308	13.12	0.000293	7/29/15	19/88/143	*PCNT*
Dominant	3	rs140685	15	24771205	4.14	0.0419	7.867	0.005	0.7902	0.374	0.374	10.14	0.001451	4/13/34	21/125/107	*GABRA5*
Recessive	1	rs1339847	1	246105917	11.66	0.0006	6.062	0.0138	0.2731	0.6013	0.6747	13.84	0.000199	6/11/34	4/65/184	*TRIM58*
Recessive	2	rs6733871	2	80383467	5.553	0.0185	6.125	0.0133	2.058	0.1514	0.5334	12.24	0.000469	18/18/15	37/128/88	*LRRTM1*
Recessive	3	rs913588	9	7164673	4.14	0.0419	6.062	0.0138	1.816	0.1778	0.5334	10.91	0.000956	4/11/36	2/55/196	*JMJD2C*
Recessive	4	rs1079109	1	159761664	3.951	0.0469	6.529	0.0106	NA	NA	NA	10.16	0.001432	3/8/38	1/93/156	*HSPA6*
Recessive	5	rs11088981	21	43694578	3.991	0.0458	5.919	0.015	1.816	0.1778	0.5334	9.754	0.00179	3/14/34	1/55/195	*C21orf125*
Recessive	6	rs3779234	7	35676367	7.646	0.0057	8.992	0.0027	0.9782	0.3226	0.6747	9.313	0.002276	8/14/29	11/116/126	*HERPUD2*
Recessive	7	rs12831803	12	124127104	3.991	0.0458	4.344	0.0371	1.816	0.1778	0.5334	8.362	0.003831	4/11/36	3/81/169	*AACS*
Recessive	8	rs2032887	19	8027360	4.14	0.0419	4.344	0.0371	NA	NA	NA	8.362	0.003831	4/13/34	3/61/189	*CCL25*
Recessive	9	rs12609976	19	60279634	3.991	0.0458	6.062	0.0138	0.7278	0.3936	0.6747	6.803	0.009103	3/9/39	2/54/197	*EPS8L1*
Recessive	10	rs7173826	15	65315428	5.157	0.0232	5.143	0.0234	0.4055	0.5243	0.6747	5.549	0.01849	12/15/23	29/124/99	*FLJ11506*
Recessive	11	rs2070180	3	122834028	3.991	0.0458	5.99	0.0144	0.1762	0.6747	0.6747	5.508	0.01893	2/5/43	1/47/204	*HCLS1*
Recessive	12	rs10907376	1	221634426	3.991	0.0458	4.344	0.0371	0.1879	0.6647	0.6747	4.839	0.02782	3/11/37	3/50/200	*C1orf65*
Recessive	13	rs6667999	1	223600307	3.999	0.0455	4.455	0.0348	0.4701	0.493	0.6747	3.921	0.04769	15/26/10	44/136/73	*DNAH14*
Recessive	14	rs316019	6	160590272	3.991	0.0458	6.935	0.0085	0.582	0.4455	0.6747	3.467	0.0626	3/10/38	4/54/194	*SLC22A2*

CHR, chromosome number; Position, chromosomal position (bp); *Q*, *Q* value for FDR correction of multiple comparison; Related gene, the nearest gene from the SNP site; NA, data not available.

**Table 5:** Top candidate SNPs selected after second stage analysis in 3-stage GWAS targeting nonsynonymous SNPs (dysesthesia)

**Model**	**Rank**	**SNP**	**CHR**	**Position**	**1st stage**		**2nd stage**		**Final stage**			**Combined**		**Genotype**		**Related gene**
					***X***^***2***^	***P***	***X***^***2***^	***P***	***X***^***2***^	***P***	***Q***	***X***^***2***^	***P***	**Abnormal**	**Normal**	
Trend	1	rs2677879	18	2537500	4.078	0.0435	6.071	0.0137	6.585	0.0103	0.0309*	16.56	4.72E-05	13/51/84	28/73/51	*METTL4*
Trend	2	rs3803800	17	7403693	7.797	0.0052	4.983	0.0256	0.2319	0.6301	0.6301	7.157	0.007467	20/73/56	6/75/73	*TNFSF13*
Trend	3	rs3777722	6	167272094	7.531	0.0061	1.063	0.3025	1.063	0.3025	0.4538	4.367	0.03665	20/68/60	11/66/77	*RNASET2*
Dominant	1	rs2677879	18	2537500	3.905	0.0481	5.79	0.0161	6.669	0.0098	0.0491*	16.31	5.37E-05	13/51/84	28/73/51	*METTL4*
Dominant	2	rs1047406	8	22626880	7.581	0.0059	4.356	0.0369	0.4674	0.4942	0.4942	10.69	0.001078	10/52/87	13/80/61	*PEBP4*
Dominant	3	rs11205415	1	247087307	4.237	0.0396	4.381	0.0363	1.34	0.2471	0.4118	10.17	0.00143	23/68/58	28/92/34	*LOC727776*
Dominant	4	rs2240308	17	60985053	4.246	0.0393	5.061	0.0245	0.7561	0.3845	0.4806	8.659	0.003254	16/74/59	16/51/87	*AXIN2*
Dominant	5	rs3777722	6	167272094	4.439	0.0351	6.171	0.013	3.221	0.0727	0.1818	2.725	0.09881	20/68/60	11/66/77	*RNASET2*
Recessive	1	rs3803800	17	7403693	5.97	0.0146	6.008	0.0142	0.0048	0.9445	0.9445	8.762	0.003076	20/73/56	6/75/73	*TNFSF13*
Recessive	2	rs3213706	11	22837578	4.37	0.0366	7.551	0.006	0.1556	0.6932	0.9445	5.98	0.01447	8/73/68	21/58/75	*LOC645581*

CHR, chromosome number; Position, chromosomal position (bp); *Q*, *Q* value for FDR correction of multiple comparison; Related gene, the nearest gene from the SNP site; *, Significant after FDR correction (*Q* < 0.05).

### Candidate loci revealed by the GWAS were located around/within the gene regions of ARID1B, ZPLD1, and METTL4

**Figure 1:**
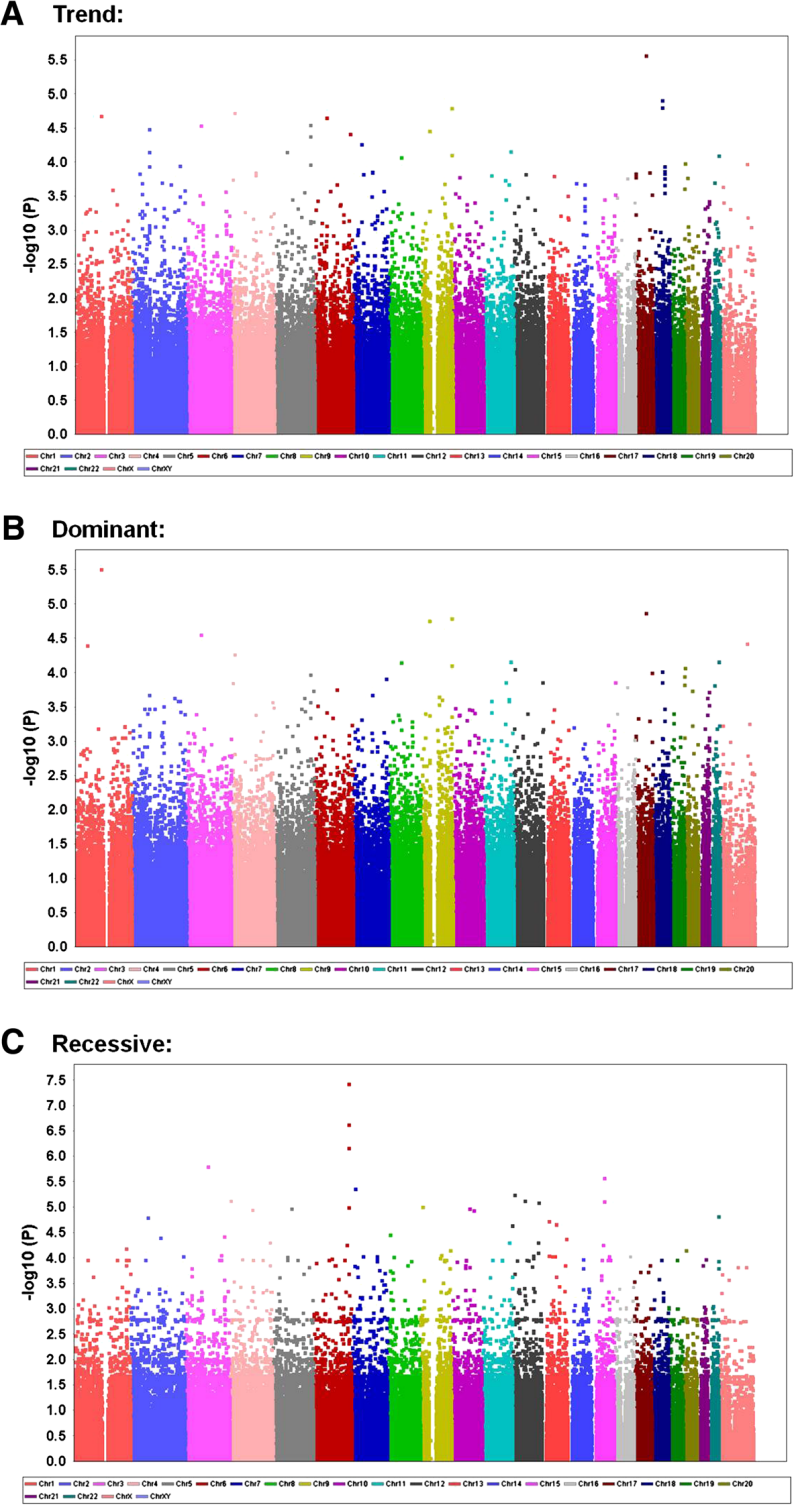
**Genome-wide association for all samples between polymorphism markers and susceptibility to hypoesthesia evaluated by the Semmes-Weinstein pressure aesthesiometer test after BSSRO in (A) trend, (B) dominant, and (C) recessive models.** The data are plotted as *–log*_*10*_ (*P* value) for each chromosome of 1-22 and X (from left to right).

**Figure 2:**
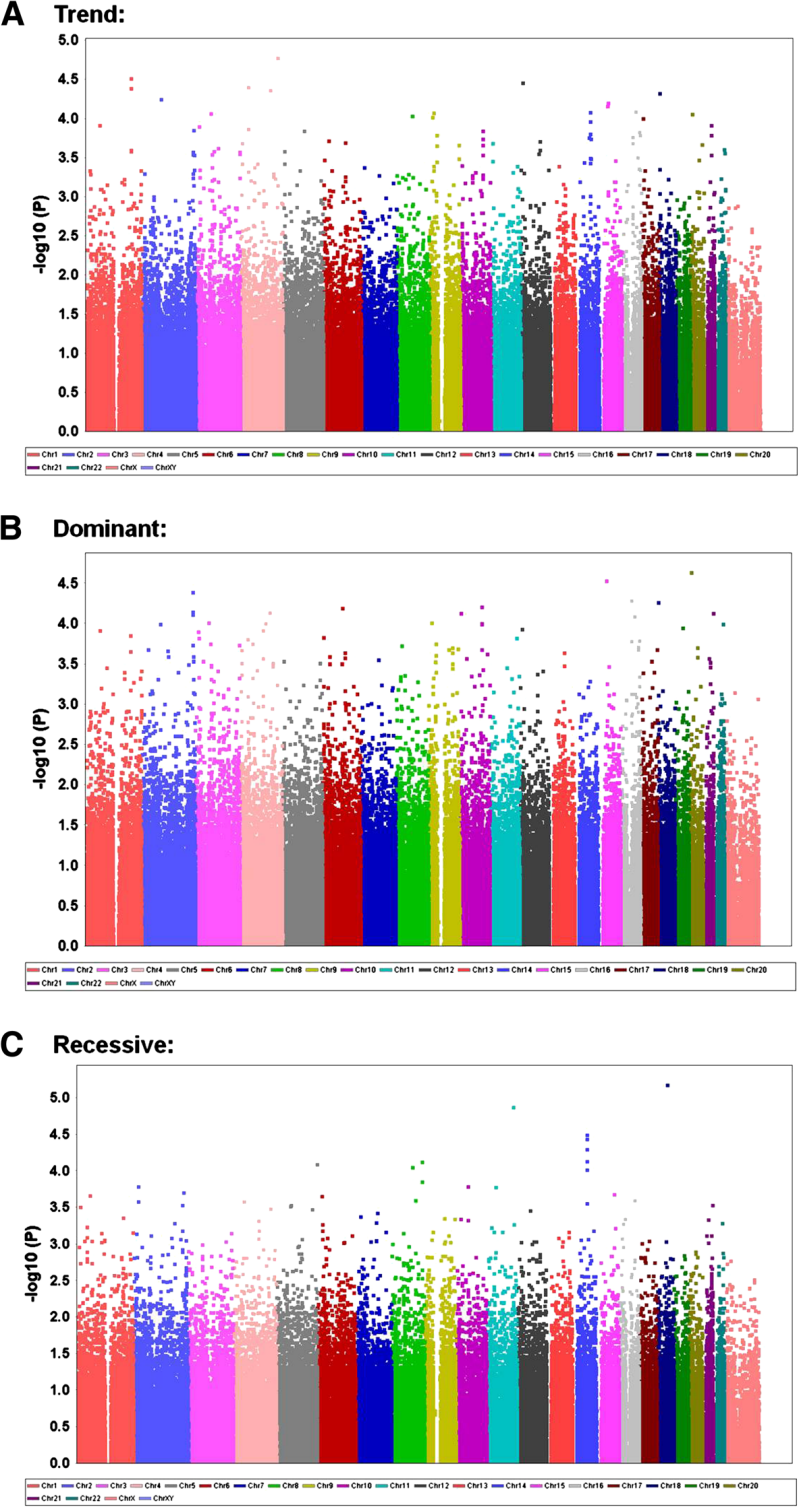
**Genome-wide association for all samples between polymorphism markers and susceptibility to dysesthesia after BSSRO in (A) trend, (B) dominant, and (C) recessive models.** The data are plotted as *–log*_*10*_ (*P* value) for each chromosome of 1-22 and X (from left to right).

Figures 1 and 2 present the genome-wide associations between polymorphism markers and the susceptibility to hypoesthesia evaluated by the Semmes-Weinstein pressure aesthesiometer test after BSSRO for all of the samples in each model for each chromosome. Significant associations were found between hypoesthesia and the rs502281 SNP (recessive model: combined *χ*^*2*^ = 24.72, nominal *P* = 6.633 × 10^-7^; Table 2; Additional file 3: Table S1) and rs2063640 SNP (recessive model: combined *χ*^*2*^ = 23.07, nominal *P* = 1.563 × 10^-6^; Table 2; Additional file 3: Table S2) and between dysesthesia and the rs2677879 SNP (trend model: combined *χ*^*2*^ = 16.56, nominal *P* = 4.722 × 10^-5^; dominant model: combined *χ*^*2*^ = 16.31, nominal *P* = 5.369 × 10^-5^; Table 5; Additional file 3: Table S3) in two independent patterns of analyses with all of the samples. According to the annotation information supplied by the manufacturer of the whole-genome genotyping arrays (Illumina, San Diego, CA), the rs502281 and rs2063640 SNPs are located within the gene flanking region of *ARID1B* and *ZPLD1* on chromosomes 6 and 3 (Table 2; Figure 1), whose official names are “AT rich interactive domain 1B (SWI1-like)” and “zona pellucida-like domain containing 1”, respectively, based on the National Center for Biotechnology Information database [10]. The rs2677879 SNP is located within the gene region of *METTL4* on chromosome 18 (Table 5; Figure 2), whose official name is “methyltransferase like 4”, based on the same database.

## Discussion

The present study explored genome-wide associations between common genetic variations and sensory disturbances after BSSRO. There are occasional reports in the literature about the relationship between individual genetic polymorphisms and neuropathic pain [11,12]. One study investigated the association between catechol-*O*-methyltransferase gene polymorphisms and pain sensitivity and musculoskeletal pain attributed to temporomandibular disorders [13]. Another study focused on the association between HLA gene polymorphisms and postherpetic neuralgia, also known as intractable chronic pain disorder [14]. Although a GWAS was previously conducted in patients with neuropathic pain induced by administration of paclitaxel for breast cancer [15], no other such studies have been performed to determine the development of postoperative peripheral neuropathy. BSSRO is among the most frequent surgical procedures in the area of oral surgery, and its procedures are well standardized. Because patient candidates for BSSRO are relatively healthy and young, they are a good population for studies of postoperative peripheral neuropathy. We conducted a GWAS to investigate the onset of sensory disturbances after BSSRO.

The results of the present study showed that hypoesthesia and dysesthesia occurred in 16.8% (51 of 304) and 49.2% (149 of 303) of the patients, respectively. Our incidence rate for hypoesthesia tended to be lower than previously reported incidences that ranged from 25% to 56% [3-5]. One reason for this may be the fact that BSSRO is performed by a limited number of skilled surgeons at our hospital, although several other reasons may explain the lower incidence of hypoesthesia. Hypoesthesia and dysesthesia are classified into vulnerability of the peripheral nerve to external stress and property of emergence of neuropathic pain following nerve injury, respectively. Thereby, the candidate genes, which were found in the present study, should be associated with these two aspects.

The GWAS identified *ARID1B*, *ZPLD1*, and *METTL4* as candidates that may be associated with the onset of sensory disturbances. The *ARID1B* gene, which is located in 6q25.3, encodes a protein that is a member of the ARID family of DNA-binding proteins and a subunit of human SWI/SNF-related complexes. The SWI/SNF complexes are known to use energy generated by an integral adenosine triphosphatase subunit to remodel chromatin. These complexes are involved in maintaining normal cellular functions and restricting the access of regulatory factors to nucleosomal DNA [16]. The *ARID1B* gene has been suggested to be associated with the occurrence of Coffin-Siris syndrome [17], a multiple congenital anomaly/mental retardation syndrome characterized by mild to moderate mental retardation, moderate to severe hypotonia, epilepsy, and congenital malformation, including a coarse facial appearance and incompletely formed fifth fingers and toes. Haploinsufficiency of the *ARID1B* gene is speculated to be a common potential cause of intellectual disability and speech impairment. The nervous system may be involved in the intractability and chronicity of neuropathic pain [18,19], but it is unclear whether *ARID1B* is associated with pain mechanism. According to the HapMap database [20], however, the rs2817461 and rs502281 SNPs identified in the present study are located upstream (approximately 200 kbp) from the *ARID1B* gene. Further studies are needed to examine the effects of these SNPs on *ARID1B* gene expression and function.

The functions of ZPLD1 remain unclear, but one report investigated the involvement of ZPLD1 in cerebral cavernous malformations [21]. The *ZPLD1* gene may be involved in the development of cerebral cavernous malformations at the mRNA expression level. Additionally, a high incidence of epilepsy is found in patients with cerebral cavernous malformations [22], suggesting the involvement of ZPLD1 in the nervous system. ZPLD1 is also reportedly associated with childhood obesity [23]. However, it is unclear whether *ZPLD1* is associated with pain mechanism. According to the HapMap database, the re2063640 SNP identified as a candidate in the present study is located in a relatively downstream region (approximately 4 kbp) that is close to the *ZPLD1* gene. This SNP may exert an effect on the gene expression level of ZPLD1, but this needs to be clarified in future studies.

The *METTL4* gene is located on the chromosome region 18p11.32. Detailed information on the functions of its gene product, however, is unavailable. No studies of which we are aware have reported associations between METTL4 and specific diseases. Based on the molecular structure of METTL4, it may affect methylation, which plays a major role in various epigenetic regulatory mechanisms. DNA methylation, recognized as the most common type of epigenetic modifications, is involved in gene silencing and plays an important role in gene regulation, development, and tumorigenesis. It has also been shown to be associated with the pathophysiology of various nervous and mental disorders. A mutation in MeCP2, a methyl-CpG binding protein, reportedly causes Rett syndrome, characterized by mental retardation and autism [24]. With regard to acquired mental disorders, abnormal DNA methylation is found in the brains of patients with schizophrenia and depression. Using microarray technology, Mill *et al*. comprehensively analyzed DNA methylation in the frontal lobe in patients with schizophrenia and bipolar (manic-depressive) disorder and found changes in the DNA methylation of genes involved in brain development and stress responses [25]. According to the dbSNP database [26], the rs2677879 SNP, a candidate identified in the present GWAS of nonsynonymous polymorphisms, leads to amino acid substitution from Gln to Lys, likely causing functional changes in the protein. Although the precise functions of *METTL4* are poorly understood, a representative *METTL*, *METTL11A*, reportedly exhibited catalytic activity as a histone methyltransferase [27]. Although future studies are needed, the action of *METTL4* might be involved in methyltransferase activity and thus cause the methylation of genomic DNA close to related genes, which could result in the modulation of neural transmission related to sensory disturbances.

The genes identified in the present study are different from those previously reported to be associated with neuropathic pain. Future studies that involve larger numbers of patients may identify previously reported gene polymorphisms and determine the functional relationships between the three gene polymorphisms identified as candidates in the present study and peripheral neuropathy. We did not consider the patients’ personalities (i.e., psychological factors) in the present study, which should be addressed in future studies.

## Conclusion

The present GWAS determined the onset of sensory disturbances after BBSRO and identified three gene polymorphisms in or near the region of the *ARIBD1*, *ZPLD1*, and *METTL4* genes. Elucidating the relationship between neuropathic pain and genetic factors will elucidate the risk factors for neuropathic pain in individual patients, thereby allowing the selection of tailored treatments.

## Methods

### Patients

Enrolled in the study were 304 healthy patients (American Society of Anesthesiologists Physical Status I; age, 15–50 years; 114 males and 190 females) who were scheduled to undergo BSSRO for mandibular prognathism at Tokyo Dental College Suidoubashi Hospital (Table 6). The study protocol was approved by the Institutional Review Board, Tokyo Dental College, Chiba, Japan, and the Institutional Review Board, Tokyo Institute of Psychiatry (currently Tokyo Metropolitan Institute of Medical Science), Tokyo, Japan. Written informed consent was obtained from all of the patients or parents when the patients were younger than 20 years old and any accompanying image. Patients who presented with distinct nerve injury during BSSRO were excluded from the study.

**Table 6:** Clinical data

**All patients (male, *n *= 114 ; female, *n *= 190)**	
Age (mean ± SEM) (range)	26.0 ± 7.6 years (15–50 years)
Body weight (mean ± SEM) (range)	58.0 ± 10.9 kg (40–128 kg)
Body height (mean ± SEM) (range)	164.7 ± 9.0cm (143–190 cm)
Loss of blood volume (mean ± SEM) (range)	161.0 ± 145.5ml (4–1400 ml)
Migration length of bone fragments (mean ± SEM) (range)	4.6 ± 2.7 mm (0–13 mm)
Duration of anesthesia (mean ± SEM) (range)	187 ± 71 min (107–864 min)
Duration of surgery (mean ± SEM) (range)	115 ± 45 min (66–750 min)

### Anesthesia and surgery

**Figure 3:**
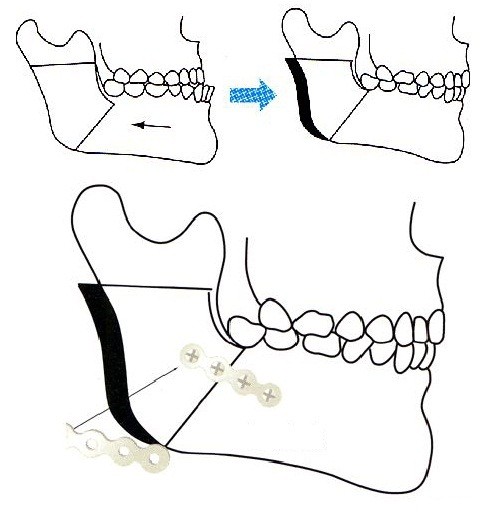
Illustration of bilateral sagittal split ramus osteotomy, which sagittally splits the mandibular ramus into inside and outside bone fragments.

Four experienced, skilled surgeons were selected. These surgeons were board-certified in the oral surgery specialty. General anesthesia was induced with target-controlled infusion (TCI) of propofol using a TCI pump (TE-371, Terumo, Tokyo, Japan). Vecuronium (0.1 mg/kg) was administered to facilitate nasotracheal intubation. After the induction of anesthesia, 10 ml of venous blood was sampled for the preparation of DNA specimens. General anesthesia was maintained with propofol at a target blood concentration of 4–6 μg/ml. Vecuronium was administered at a rate of 0.08 mg/kg/h. The lungs were ventilated with oxygen-enriched air. Local anesthesia was performed on the right side of the surgical field with 8 ml of 2% lidocaine that contained 12.5 μg/ml epinephrine, and right mandibular ramus osteotomy was performed. Local anesthesia was then performed on the left side, and left mandibular ramus osteotomy was performed. The bilateral mandibular bone segments were fixed in appropriate positions (Figure 3). Whenever systolic blood pressure or heart rate exceeded +20% of the preinduction value during surgery, intravenous (i.v.) fentanyl (1 μg/kg) was administered. At the end of surgery, a rectal diclofenac sodium suppository (50 mg) and dexamethasone (8 mg, i.v.) were administered to prevent orofacial edema and postoperative pain. Oral mecobalamin (1.5 mg/day) was administered for 4 weeks after the operation.

### Evaluation of sensory disturbances

**Figure 4:**
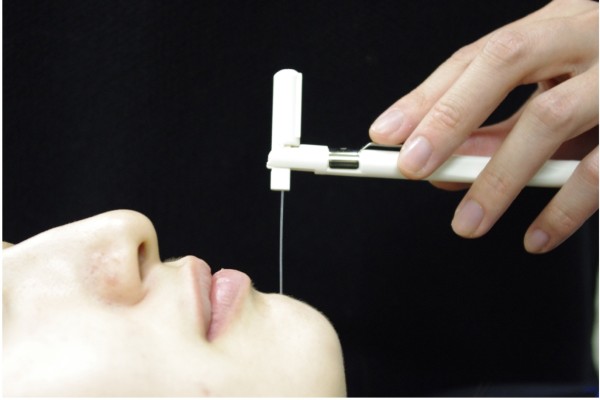
**Photograph of Semmes-Weinstein pressure aesthesiometer test, which consists of 20 individual filaments with varying diameters.** These filaments are mounted into individual Lucite rods.

Sensory disturbances were determined postoperatively by the presence of hypoesthesia or dysesthesia in the mental nerve area. Hypoesthesia was evaluated by tactile-threshold tests 1 week after the operation. The 1 week time-point was chosen for assessment to avoid testing during the time when postoperative pain was severe. The tactile-threshold test was performed using a Semmes-Weinstein pressure aesthesiometer (Research Design, Houston, TX, USA; (Figure 4) [28]. The Semmes-Weinstein pressure aesthesiometer consisted of 20 filaments with different diameters. The end of each filament was mounted into an individual Lucite rod. The amount of force applied through the long axis of each filament to achieve a noticeable bend was determined. The magnitude of these forces ranged from 0.0045 g to 447 g. This test was performed by two experienced dentists.

Touch stimulation was performed using the method of Bell [29]. The Semmes-Weinstein pressure aesthesiometer was perpendicularly lowered to a test region for 1–1.5 s and then lifted for 1–1.5 s. Stimulation was applied three times with 1.65-4.08 manufacturer’s filament marking and calculated force (Fmg) and once with 4.17-6.65 Fmg at each point. All of these filaments, with the exception of the largest (6.65 Fmg), bent when they reached the specified pressure. Stimulation began with the 1.65 Fmg filament (i.e., the thinnest filament), and the stimulation force was increased until the patient perceived the stimulation. Tactile sensitivity was recognized to be positive when the patient perceived any stimulation, even if the stimulation was not perceived as a normal tactile sensation.

Based on the running courses of the labial inferior ramification and mental ramification, measurements were performed at two points [3]: (1) the vermilion border at one-third the distance between the oral angles and (2) the midpoint of the perpendicular line from point (1) to the lower margin of the mentum.

The worst among the values obtained at the four total test-points on both sides was regarded as the representative value. This value was evaluated by the interpretation scale reported by Bell [29]. In this scale, sensory function is classified into five grades. In the present study, the patients who were classified into grades that were worse than the second grade (2.83 Fmg) were regarded as hypoesthesic.

A patient who spontaneously recognized any abnormal sensations was regarded as dysesthesic. The evaluation of dysesthesia was based on the definition of the International Association for the Study of Pain. Subjective symptoms were assessed by interview 4 weeks after the operation. The patients were asked to select words from the McGill Pain Questionnaires [30] to describe their pain (i.e., temporal, brightness, thermal, dullness, traction pressure, constrictive pressure, etc.). The time-point of 4 weeks was chosen for assessment to avoid testing during the time of Wallerian [31] degeneration and retrograde degeneration after nerve damage.

### Whole-genome genotyping

Genomic DNA was extracted from whole-blood samples using standard procedures. The extracted DNA was dissolved in TE buffer (10 mM Tris–HCl and 1 mM ethylenediaminetetraacetic acid, pH 8.0). The DNA concentration was adjusted to 100 ng/μl using a NanoDrop ND-1000 Spectrophotometer (NanoDrop Technologies, Wilmington, DE, USA).

Whole-genome genotyping was performed using Infinium assay II utilizing an iScan system (Illumina) according to the manufacturer’s instructions, with a total of 361 samples including those of the patients enrolled in the study. Genotyping was conducted basically the same way as a previous report [32]. Five kinds of BeadChips were used for genotyping 40, 67, 6, 120, and 128 samples, respectively: HumanHap300 (total markers: 317,503), HumanHap300-Duo (total markers: 318,237), Human610-Quad v1 (total markers: 620,901), Human1M v1.0 (total markers: 1,072,820), and Human 1M-Duo v3 (total markers: 1,199,187). Some BeadChips include a number of probes that are specific to copy number variation markers, but most were for SNP markers on the human autosome or sex chromosome. Approximately 300,000 SNP markers were commonly included in all of the BeadChips.

### Quality control

The data for the genotyped samples were analyzed using BeadStudio or GenomeStudio with the Genotyping module v3.3.7 (Illumina) to evaluate the quality of the results. The genotype data from all five of the BeadChips were merged to analyze all of the samples simultaneously (i.e., only the markers common to all of the BeadChips were included in the analysis, and the others were automatically excluded). In the data-cleaning process, the samples with a genotype call rate of less than 0.95 were excluded from further analyses. Markers with a genotype call frequency of less than 0.95, “Cluster sep” (i.e., an index for genotype cluster separation) of less than 0.1, and minor allele frequencies of less than 0.05 were excluded from the subsequent association study.

### Statistical analysis

Prior to the GWAS, associations between the clinical data and hypoesthesia or dysesthesia expression frequency after BSSRO were analyzed. Clinical data included gender, age, body weight, body height, loss of blood volume, migration length of bone fragments, duration of anesthesia, and duration of surgery (Table 6). A logistic regression analysis was performed using SPSS (12.0J for Windows, SPSS Japan, Tokyo, Japan).

The Fisher’s exact test was performed for all of the genotype frequency data to investigate the deviation of the distributions from those in the theoretical Hardy-Weinberg equilibrium, which sometimes reflects genotyping errors or population stratification of the samples. Markers with *P* values (*df* = 1) greater than approximately 2 × 10^-7^ (0.05/300,000) were considered for the GWAS.

A multistage GWAS was conducted for the patients who underwent painful cosmetic surgery to investigate the association between genetic variations and sensory disturbances after BSSRO. Among 361 subjects, one subject did not meet the quality control criteria in our preliminary analysis, and 57 and 58 subjects lacked clinical data for hypoesthesia and dysesthesia, respectively. Therefore, genotype data for a total of 360 subjects were used for our three-stage GWAS (120 subjects for each of the first-, second-, and final-stage analyses). Clinical data for a total of 304 and 303 subjects were used for our three-stage GWAS of hypoesthesia (104, 98, and 102 subjects for the first-, second-, and final-stage analyses, respectively) and dysesthesia (105, 96, and 102 subjects for the first-, second-, and final-stage analyses, respectively), respectively. The subjects were recruited within several years and randomly categorized into three independent groups to minimize bias in the clinical data, indicating that the samples and clinical data were not used in chronological order for our first-, second-, and final-stage analyses. In our preliminary analysis that used merged markers between different BeadChips with BeadStudio or GenomeStudio, 295,036 SNPs (including 6,016 nonsynonymous SNPs) were selected for the analyses.

For the GWAS, the Cochran-Armitage trend test was performed to explore markers that might confer susceptibility to hypoesthesia evaluated by the Semmes-Wemstem pressure aesthesiometer test or dysesthesia after BSSRO. The patients were divided into two groups based on the presence or absence of symptoms, and a linear trend analysis of the increased rate of subjects with an increased number of variant risk alleles was performed for all markers. Moreover, dominant and recessive genetic models for each minor allele were used for the analyses because of the previously insufficient knowledge about the genetic factors associated with sensory disturbances after BSSRO. The association study included both female and male subjects for autosomal markers, although male genotypes were excluded from the analysis of X chromosome markers. All of the statistical analyses were performed using gPLINK v. 2.050, PLINK v. 1.07 PLINK [33], and Haploview v. 4.1 [34]. Single-nucleotide polymorphism annotations were created based on an annotation file within Human 1M-Duo v3 supplied by the manufacturer of the BeadChips. For calculation of *Q*-values, SFDR (Stratified False Discovery Rate) software [35] or PLINK v. 1.07 was used. Power analyses were performed using G*Power v. 3.0.5 [36].

The GWAS procedure is summarized in the Additional file 1: Figure S1 and Additional file 2: Figure S2. In the first-stage analysis of 104 and 105 subjects for hypoesthesia and dysesthesia, respectively, the SNPs that had statistical *P* values of less than 0.05 were selected as the candidate SNPs for the second-stage analysis among the SNP that passed the quality control criteria within the 295,036 SNPs (6,016 nonsynonymous SNPs). For these SNPs, the second-stage analysis was conducted. Again, the SNPs that had *P* values of less than 0.05 were considered potential candidates and selected for further final-stage analysis. Linkage disequilibrium (LD)-based SNP pruning was also conducted in this stage utilizing PLINK v. 1.07 software, and SNPs that were in approximate linkage equilibrium with an SNP were excluded based on the following process: (*i*) consider a window of 50 SNPs, (*ii*) calculate LD between each pair of SNPs in the window, (*iii*) remove one of a pair of SNPs if the LD is greater than 0.8, and (*iv*) shift the window five SNPs forward and repeat the procedure. In the final stage, the association study was conducted to determine whether the possible associations between the SNPs selected in the second stage and phenotypic traits would be strictly replicated. In this stage, the *Q* values of the false discovery rate were calculated to correct for multiple testing, in addition to *P* values based on previous reports [37,38]. The SNPs with *Q* < 0.05 in the analysis were considered genome-wide significant.

Two independent patterns of the GWAS were conducted to effectively explore candidate SNPs that showed statistically strong association with the phenotypic traits and those that could functionally impact neighboring genes. In the first pattern, a normal GWAS procedure targeted all of the SNPs that were available (Additional file 1: Figure S1). In the second pattern, the GWAS procedure targeted only nonsynonymous SNPs that tended to affect the function of the protein encoded by the relevant gene (Additional file 2: Figure S2).

A log quantile-quantile (QQ) *P*-value plot as a result of the GWAS for the combined samples was subsequently drawn to check the pattern of the generated *P*-value distribution, in which the observed *P* values against the values expected from the null hypothesis of uniform distribution, calculated as –log10 (*P* value), were plotted for each model. Many of the plots were mostly concordant with the expected line (*y* = *x*), especially over the range of 0 < −log10 (*P* value) < 4, indicating no apparent population stratification of the samples used in the study, although the plots for the recessive model, especially for hypoesthesia, apparently deviated over the range of –log10 (*P* value) > 3 (Additional file 4: Figure S3 and Additional file 5: Figure S4).

## Abbreviations

GWAS: Genome-wide association study; BSSRO: Bilateral sagittal split ramus osteotomy; TCI: Target-controlled infusion; QQ: Quantile-quantile.

## Competing interests

The authors declare that they have no competing interests.

## Authors’ contributions

DK conceived the study, analyzed the data, generated the figures, and contributed to writing the manuscript. DN conceived the study, performed the molecular genetic studies, analyzed the data, generated the figures, and contributed to writing the manuscript. YT and TK performed most of the operations on the patients in this study and analyzed the data. SK performed the molecular genetic studies and conceived the study. KI and KF participated in conceiving the design, analyzed the data, and edited the manuscript. All the authors read and approved the final manuscript.

## Supplementary Material

Additional file 1: Figure S1 Schematic illustration of the multistage GWAS that targeted all of the SNPs that were available. Potent candidate SNPs associated with (**A**) hypoesthesia evaluated by the Semmes-Weinstein pressure aesthesiometer test and (**B**) dysesthesia after BSSRO were selected for the three-stage GWAS. Click here for file

Additional file 2: Figure S2 Schematic illustration of the multistage GWAS that targeted only nonsynonymous SNPs. Potent candidate SNPs associated with (**A**) hypoesthesia evaluated by the Semmes-Weinstein pressure aesthesiometer test and (**B**) dysesthesia after BSSRO were selected for the three-stage GWAS. Click here for file

Additional file 3 **Table S1.** Frequencies of hypoesthesia in patients with the *ARID1B* (rs502281) genotype. **Table S2** Frequencies of hypoesthesia in patients with the *ZPLD1* (rs2063640) genotype. **Table S3** Frequencies of dysesthesia in patients with the *METTL4* (rs2677879) genotype. Click here for file

Additional file 4: Figure S3 Log quantile-quantile (QQ) *P* value plot for all of the samples as a result of the GWAS for hypoesthesia evaluated by the Semmes-Weinstein pressure aesthesiometer test after BSSRO in (**A**) trend, (**B**) dominant, and (**C**) recessive models. Click here for file

Additional file 5: Figure S4 Log quantile-quantile (QQ) *P* value plot for all of the samples as a result of the GWAS for dysesthesia after BSSRO in (**A**) trend, (**B**) dominant, and (**C**) recessive models. Click here for file
