# Supplementary figures and images for: Genome-wide association study of sensory disturbances in the inferior alveolar nerve after bilateral sagittal split ramus osteotomy
Source: Mol Pain. 2013 Jul 8;9:34. doi: 10.1186/1744-8069-9-34 (PMC3723511; doi:10.1186/1744-8069-9-34)

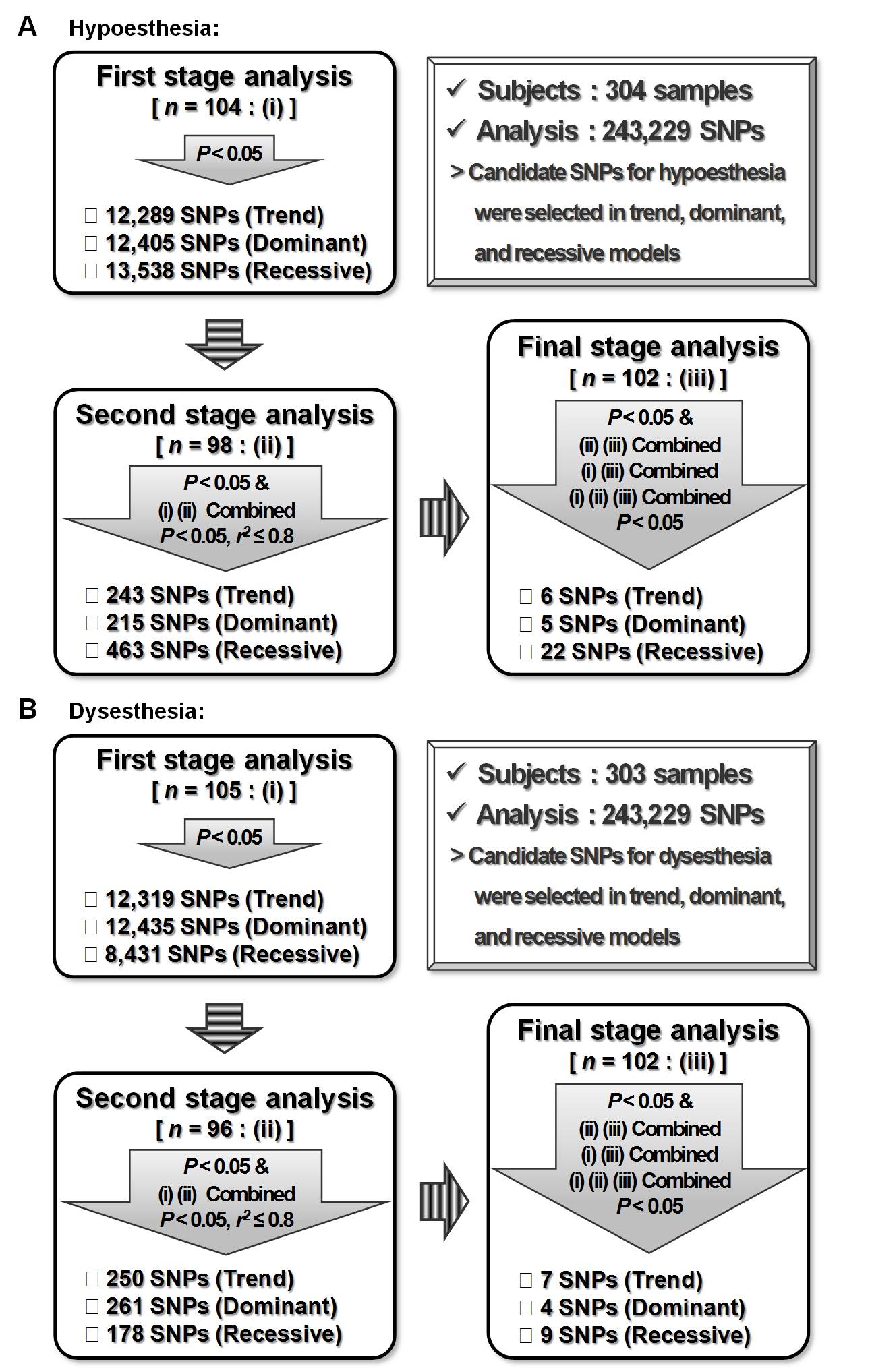

Supplement: Additional file 1: Figure S1 — Schematic illustration of the multistage GWAS that targeted all of the SNPs that were available. Potent candidate SNPs associated with (A) hypoesthesia evaluated by the Semmes-Weinstein pressure aesthesiometer test and (B) dysesthesia after BSSRO were selected for the three-stage GWAS. [file 1744-8069-9-34-S1.jpeg]

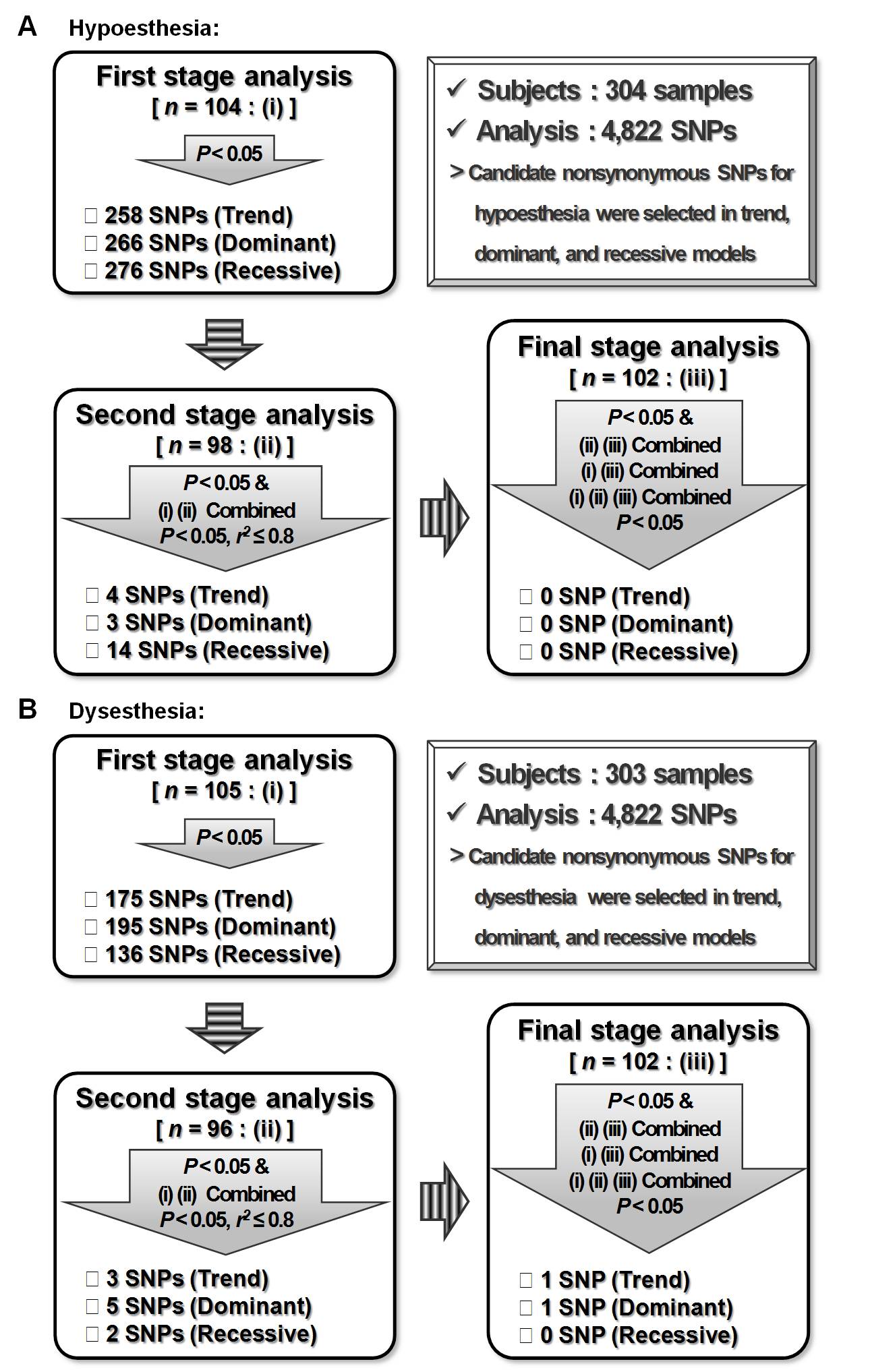

Supplement: Additional file 2: Figure S2 — Schematic illustration of the multistage GWAS that targeted only nonsynonymous SNPs. Potent candidate SNPs associated with (A) hypoesthesia evaluated by the Semmes-Weinstein pressure aesthesiometer test and (B) dysesthesia after BSSRO were selected for the three-stage GWAS. [file 1744-8069-9-34-S2.jpeg]

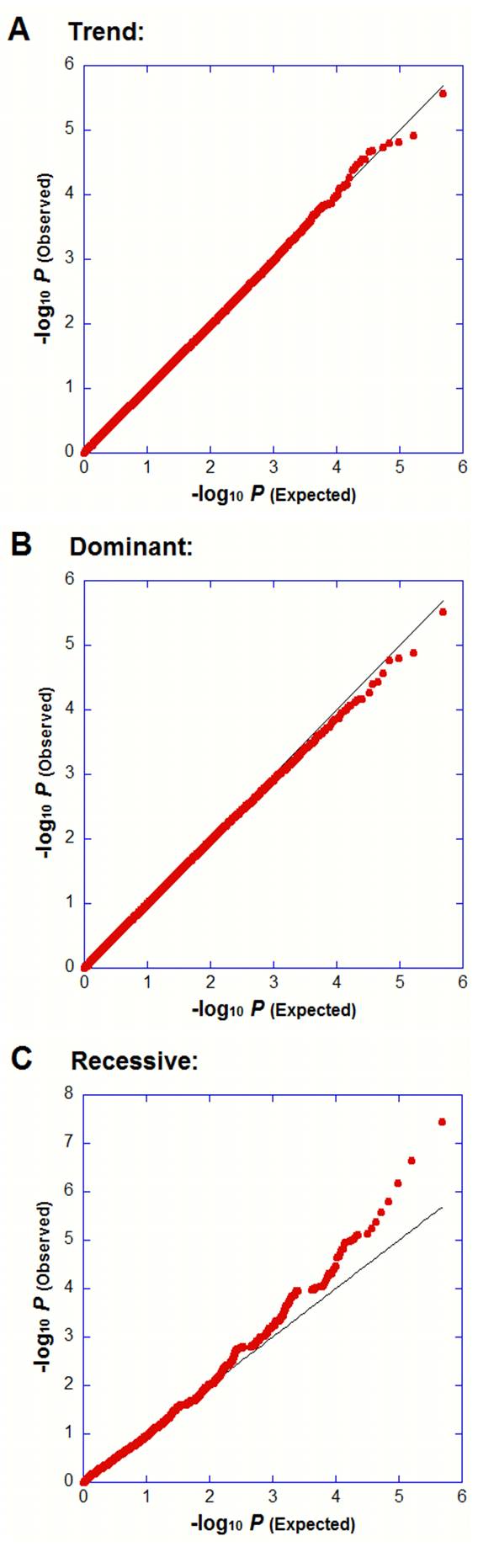

Supplement: Additional file 4: Figure S3 — Log quantile-quantile (QQ) P value plot for all of the samples as a result of the GWAS for hypoesthesia evaluated by the Semmes-Weinstein pressure aesthesiometer test after BSSRO in (A) trend, (B) dominant, and (C) recessive models. [file 1744-8069-9-34-S4.jpeg]

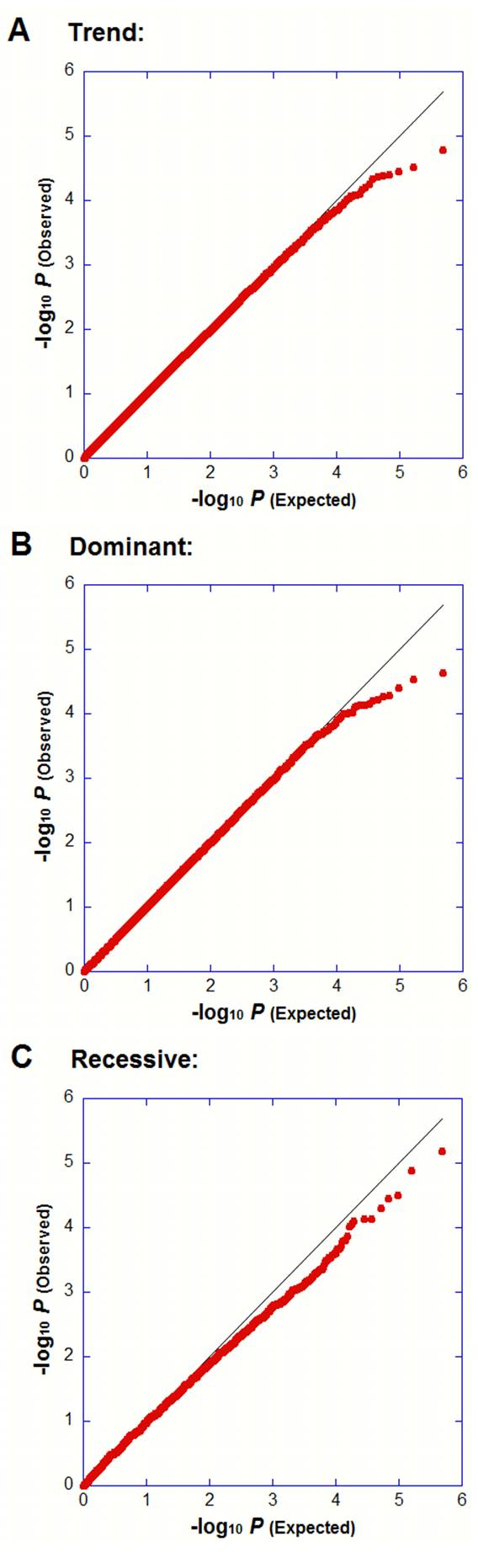

Supplement: Additional file 5: Figure S4 — Log quantile-quantile (QQ) P value plot for all of the samples as a result of the GWAS for dysesthesia after BSSRO in (A) trend, (B) dominant, and (C) recessive models. [file 1744-8069-9-34-S5.jpeg]
