# Supplementary material for: Genome-wide association study of sensory disturbances in the inferior alveolar nerve after bilateral sagittal split ramus osteotomy
Source: Mol Pain. 2013 Jul 8;9:34. doi: 10.1186/1744-8069-9-34 (PMC3723511; doi:10.1186/1744-8069-9-34)
Supplement: Additional file 3 — Table S1. Frequencies of hypoesthesia in patients with the ARID1B (rs502281) genotype. Table S2 Frequencies of hypoesthesia in patients with the ZPLD1 (rs2063640) genotype. Table S3 Frequencies of dysesthesia in patients with the METTL4 (rs2677879) genotype. [file 1744-8069-9-34-S3.docx]

**Table S1. Frequencies of hypoesthesia in patients with the *ARID1B* (rs502281) genotype.**

| Genotype | *n* | Hypoesthesia (*n*) | Frequency |
| --- | --- | --- | --- |
| CC+TC | 295 | 44 | 14.9% |
| TT | 9 | 7 | 77.8% |

**Table S2. Frequencies of hypoesthesia in patients with the *ZPLD1* (rs2063640) genotype.**

| Genotype | *n* | Hyposthesia (*n*) | Frequency |
| --- | --- | --- | --- |
| AA | 32 | 15 | 46.9% |
| AC+CC | 271 | 36 | 13.3% |

**Table S3. Frequencies of** **dysesthesia in patients with the *METTL4* (rs2677879) genotype.**

| Genotype | *n* | Dysesthesia (*n*) | Frequency |
| --- | --- | --- | --- |
| AA | 41 | 13 | 31.7% |
| AC | 124 | 51 | 41.1% |
| CC | 135 | 84 | 62.2% |
